# Genetic correction of serum AFP level improves risk prediction of primary hepatocellular carcinoma in the Dongfeng–Tongji cohort study

**DOI:** 10.1002/cam4.1481

**Published:** 2018-04-25

**Authors:** Ke Wang, Yansen Bai, Shi Chen, Jiao Huang, Jing Yuan, Weihong Chen, Ping Yao, Xiaoping Miao, Youjie Wang, Yuan Liang, Xiaomin Zhang, Meian He, Handong Yang, Huan Guo, Sheng Wei

**Affiliations:** ^1^ Department of Epidemiology and Biostatistics Ministry of Education Key Laboratory of Environment and Health School of Public Health Tongji Medical College Huazhong University of Science and Technology Wuhan Hubei China; ^2^ Department of Occupational and Environmental Health School of Public Health Tongji Medical College Huazhong University of Science and Technology Wuhan Hubei China; ^3^ Department of Nutrition and Food Hygiene School of Public Health Tongji Medical College Huazhong University of Science and Technology Wuhan Hubei China; ^4^ Department of Maternal and Child Health School of Public Health Tongji Medical College Huazhong University of Science and Technology Wuhan Hubei China; ^5^ Department of Social Medicine and Health Management School of Public Health Tongji Medical College Huazhong University of Science and Technology Wuhan Hubei China; ^6^ Dongfeng Central Hospital Dongfeng Motor Corporation and Hubei University of Medicine Shiyan Hubei China

**Keywords:** Alpha‐fetoprotein, genetic correction, prediction efficiency, primary hepatocellular carcinoma, single‐nucleotide polymorphisms

## Abstract

Serum alpha‐fetoprotein (AFP) is the most commonly used tumor biomarker for screening and diagnosis of primary hepatocellular carcinoma (HCC). However, the predictive effect for HCC risk is still unsatisfactory. The aim of this prospective study was to estimate whether the individual genetic correction could improve the prediction efficiency of AFP for HCC risk. A prospective analysis with 9819 baseline HCC‐free individuals based on a large population‐based Chinese cohort study was performed. Two single‐nucleotide polymorphisms (SNPs) associated with serum AFP level were used to calculate the genetic corrected AFP level (rs12506899 and rs2251844). Statistical analysis including logistic regression analysis and the area under the receiver operating characteristic (ROC) curve were used to assess the discriminative ability of the original and genetic corrected AFP level for HCC risk. The odds ratios (ORs) and 95% confidence intervals (95% CIs) were presented. Fifty‐seven participants were diagnosed with HCC for the first time. After adjusting AFP level with genetic effects, the participants for HCC risk increased compared to those with AFP level alone (OR = 5.34, 95% CI = 2.57–11.13; *P *<* *0.001 vs. OR = 5.04, 95% CI = 2.46–10.30; *P *<* *0.001). In addition, the area under the curve (AUC) for the discrimination of HCC elevated from 0.611 to 0.726. The efficiency in HCC prediction using serum AFP level can be improved by adjusting AFP level based on genetic effects. The genetic correction effect on serum AFP should be considered in the clinic application of such tumor biomarkers.

## Introduction

Primary hepatocellular carcinoma (HCC) is the third most frequent cause of cancer death in the world, and the associated morbidity and mortality have continued to rise each year 1. There is a high prevalence of HCC in Asia, particularly in China 2. The time‐to‐diagnosis plays a decisive role in disease progression, effectiveness of early treatment, and five‐year survival rate of HCC 3. A negative finding based on the current WHO diagnostic criteria for HCC does not conclusively rule out a diagnosis of HCC, as the false‐negative rate can approach 30% due to sampling errors, this is in part due to the absence of specific histological markers for HCC 4. Consequently, it is particularly important to early prediction the risk of HCC.

The use of blood‐based biomarkers has been widely considered as a non‐invasive tool for early diagnosis of HCC, based on convenience, their quantitative nature, and cost‐effectiveness 5. Among them, alpha‐fetoprotein (AFP) is one of the most widely used tumor biomarkers and has been in clinical use since the 1970s 6, 7. Furthermore, among serologic tests for HCC surveillance, serum AFP level is the best‐studied index and is the only serum biomarker that has undergone all phases of biomarker development 8. AFP levels are elevated in the fetus, and decline rapidly after birth, reaching their lowest levels between 6 and 8 months of age during pregnancy. AFP is nearly undetectable in healthy individuals but appears to be elevated in HCC 9. Although serum AFP is a well‐accepted tumor biomarker for HCC, serum levels of AFP are not elevated in 10–30% of HCC patients 10. However, the dynamic range in AFP levels varies considerably from one patient to another 11. Serum AFP level may also be elevated due to underlying conditions other than HCC, including nonseminomatous germ cell tumors 12. As a result, it is difficult to diagnose HCC with both high sensitivity and specificity using serum AFP level. Previous studies have shown that the efficiency in predicting HCC based only on serum AFP level (30–62%) remains unsatisfactory, with the efficiency for poorly differentiated HCC being <50% 13. One study has reported that the sensitivity of AFP for HCC in an elderly population was approximately 41.0%, while another study determined it to be 60.5% 14, 15. Therefore, the application of AFP is limited in practice, and it is necessary to find new indicators to aid the development novel predictive approaches. It could be more helpful if these indicators can be used to show the difference of individuation. However, recent studies have reported that the serum concentration of tumor biomarkers can be affected by hereditary variation 16, 17. For example, the prediction accuracy of PSA could be improved significantly after genetic correction of serum PSA compared to the raw serum PSA levels 18. It suggests that such variants could provide an individual estimate of tumor marker level. We assume that it could be improved the efficiency of HCC prediction using serum AFP by adjusting with genetic variations.

Several single‐nucleotide polymorphisms (SNPs) were found to be associated with the serum level of AFP from a genome‐wide association study we performed before 19. We propose that these variants could be account for part of variability of serum AFP levels in individuals. It may be possible to improve the efficiency in predicting HCC based on serum AFP by adjusting the serum AFP with genetic effects.

Here, to explore this hypothesis, we performed a cohort study on the value of genetic correction of AFP level for the prediction of HCC risk in a Chinese population. We aim to evaluate whether the individual level of AFP could improve the prediction efficiency for HCC relative to the prediction efficiency based only on the original AFP level.

## Materials and Methods

### Study population

All the participants in this study come from Dongfeng–Tongji cohort (DFTJ cohort). The details about this cohort have been described previously 20. The DFTJ cohort study was launched in 2008, included 27,009 people who were retired from a state‐owned automobile enterprise in 2008 in Hubei, China. All the participants were followed at baseline from September 2008 to June 2010. The information about lifestyle (drinking status, smoking status etc.), demographics, environmental and professional exposures as well as laboratory data were collected at baseline.

The genotypes of AFP‐related SNPs were obtained from a genome‐wide association study, two SNPs (rs12506899 in *AFP* and rs2251844 in *HISPPD2A*) were finally found to be related with serum AFP level 19. A total of 10,197 healthy individuals from DFTJ cohort study who had not been diagnosed with cancer or chronic diseases before enrolled in this cohort at baseline with genotyping information of these two SNPs were included in this study.

The participants in this study were from previous genome‐wide association study in DFTJ cohort (*n* = 10,197). Among them, 378 people were not able to get information about the follow‐up. In all, 9819 subjects were included in this study for data analysis.

### Measurement of serum AFP level

All the participants had health examinations after an overnight fasting until the following morning at Dongfeng Central Hospital. All of the tests were carried out by experienced and professional staff. Fifteen milliliters of fasting blood for every people was collected in the coagulation tubes. All samples were checked for accuracy of personal information and were tracked using an electronic database. AFP levels were determined by immunoassay at the laboratory of the Dongfeng Central Hospital. Serum levels of AFP were measured with an Architect Ci8200 automatic analyzer (Abbott Laboratories, Abbott Park, IL) using Abbott Diagnostics reagents according to the manufacturer's instructions, assays at baseline were all performed in the same laboratory. All samples were randomized for testing and blinded to the experimenters prior to interpretation. Values lower than the detection limit of the assay were given a “low” value of 0.005 ng/mL.

### Follow‐up and the diagnosis of primary hepatocellular carcinoma

The information about cancer incidence and deaths were confirmed with the unique medical insurance number for each participant from Dongfeng Medical Insurance Center's health‐care service system. The records of physical examination and questionnaire interview were also used to determine disease status and deaths. Electronic medical records in the Dongfeng Central Hospital were provided to us along with outpatient records and contact with inpatients to our database for further study. Major diseases including cancer, stroke, and diabetes were verified through reviews of medical records of the Dongfeng Central Hospital, which allowed us to obtain information on diseases and documentation of deaths in the follow‐up. The definition of endpoint in this study is the incident of primary hepatocellular carcinoma. The latest follow‐up of cancer in the DFTJ cohort was completed on December 31, 2016.

The diagnosis of primary hepatocellular carcinoma in this study was based on worldwide standards. The inclusion and exclusion criteria of new cases in this study were as follows: first, the diagnosis of primary hepatocellular carcinoma diagnosis was consistent with histological diagnostic criteria of the WHO 21, 22; second, the patients who were diagnosed with metastatic hepatocellular carcinoma were excluded in this study.

### Statistical analysis

The baseline characteristics of participants in this study were presented as means ± standard deviation (mean ± SD) or medians (interquartile range) for numerical variables. Student's *t*‐test was used to evaluate the significance differences between continuous variables. Mann–Whitney *U* test was used to assess for quantitative variables, where appropriate. Categorical variables were described as counts, and the Pearson chi‐squared test was performed for comparison. Unconditional logistic regression model was conducted to evaluate the risk of HCC in multivariate analyses. The odds ratios (ORs) and 95% confidence intervals (95% CIs) were presented.

The genetic corrected AFP levels were estimated by combining the measured AFP levels with genetic relative effect. For each SNP associated with the serum AFP level which was found in our genome‐wide association study performed before, a classical linear regression was used with log‐transformed value for the standardized value, which then back‐transformed to evaluate each effect of genotype, in order to test the standardized value of each SNP. The combined genetic effect was calculated based on the genotypic effect for each SNP with a multiplicative model and then combining them 18.

In addition, the area under the receiver operating characteristic (ROC) curve (AUC) with 95% confidence interval (95% CI) was used to evaluate the discriminative ability of original and genetic corrected AFP level for HCC risk. The difference of AUC between original serum AFP level and the genetic corrected AFP level were compared by a nonparametric method 23. All two‐sided *P* values <0.05 were considered to be statistically significant. All statistical analyses were performed using the SAS version 9.4 statistical software package and Empower Stats (http://www.empowerstats.com).

## Results

### Study subjects after follow‐up

A total of 9819 baseline HCC‐free participants were included in this study. The characteristics of all the participants enrolled in baseline including demographic, and biochemical indicators are shown in Table 1. As shown, 46.9% were males, the mean age and BMI of all study participants at baseline was 62.09 ± 7.78 years and 24.33 ± 3.32 kg/m^2^, respectively. Among all the subjects included in the analyses, 30.3% were with a history of smoking and 27.2% were with a history of drinking.

**Table 1:** Baseline demographic and biochemical characteristics of all individuals in this study

Characteristic	Result^a^
Age (year)	62.09 ± 7.78
Gender	
Male	4601 (46.9)
Female	5218 (53.1)
BMI (kg/m^2^)	24.33 ± 3.32
Smoking status	
Ever	2950 (30.3)
Never	6784 (69.7)
Drinking status	
Ever	2666 (27.2)
Never	7124 (72.8)
Marriage status^b^	
Yes	8802 (89.9)
No	992 (10.1)
Education level^c^	
Yes	1016 (10.4)
No	8715 (89.6)
Physical activity	
Yes	8652 (88.5)
No	1130 (11.5)
Family history of cancer	
Yes	294 (3.0)
No	9525 (97.0)
AFP (ng/mL)	2.70 (0.98–3.90)
Genetic corrected AFP (ng/mL)	2.58 (0.86–3.72)

Several variables are inconsistent with the total number because of the absence (<1.0%). AFP, alpha‐fetoprotein; BMI, body mass index.

a The characteristics of variables are presented in the forms of number (percentage), means ± standard deviation, or medians (interquartile range).

b Married or remarried.

c College or above.

In the period between the baseline and the follow‐up, fifty‐seven people were newly diagnosed with HCC (70.2% males and 29.8% females, *P *<* *0.001). The cumulative incidence rate of HCC during the follow‐up period was 5.81 cases per 1000 people. Twenty‐three subjects with HCC had a history of drinking while thirty‐four participants were non‐drinkers. There was a significant difference in drinking history between the subjects diagnosed with HCC and those without HCC (*P *=* *0.026). A history of smoking was also significantly higher in HCC patients (*P *=* *0.026). However, there was no difference between the subjects with or without HCC with respect to physical activity history (*P *=* *0.510).

### Effect of SNPs on serum AFP levels

The estimates on the relative genotype effect for SNPs associated with serum AFP level are shown in Table 2. Consequently, there was a significant difference between genetic corrected AFP levels and AFP levels alone (*P *<* *0.001). After adjustment, the total AFP levels were estimated to be 8.7% lower than the AFP levels without adjustment (7.17 ng/mL for genetic corrected AFP levels vs. 7.85 ng/mL for the original AFP levels). Moreover, it is obvious that compared to the subjects free of HCC, the serum levels of AFP with genetic correction were higher in individuals with diagnosed HCC for the first time (3.82 ± 3.89 ng/mL vs. 2.64 ± 2.08 ng/mL, *P *<* *0.001). The same conclusion was also presented in terms of the original serum levels of AFP.

**Table 2:** The SNPs associated with serum AFP level and their relative genotype effect on AFP

SNP	Chr	Position (bp)	Allelic Frequency	Allele	Relative Allelic effect	XX effect	OO effect	OX effect
rs12506899	4	74,538,147	0.33	T	1.08	1.10	0.95	1.02
rs2251844	15	41,623,770	0.47	T	1.10	0.92	1.11	1.00

For the alleles associated with serum AFP, XX: homozygous; OO: non‐carriers; OX: heterozygous. AFP, alpha‐fetoprotein; SNP, single‐nucleotide polymorphism.

### The risk of HCC based on serum AFP levels and genetic corrected AFP levels

The associations between the serum AFP levels and the risk of HCC are presented in Table 3. The risk of HCC elevated with the increase of serum AFP levels as well as genetic corrected AFP levels. What was more, a high OR for HCC was found in baseline AFP levels adjusting for genetic effects in three different models. The risk of HCC increased after adjusting AFP levels with genetic effects, compared to the risk predicted based on original serum AFP levels. With correction of the genetic effects, per 10 ng/mL increase of corrected AFP levels could significantly increase 434% HCC risk compared to that per 10 ng/mL increase of AFP levels only increase 404% HCC risk (OR = 5.34, 95% CI = 2.57–11.13; *P *<* *0.001 vs. OR = 5.04, 95% CI = 2.46–10.30; *P *<* *0.001), in the fully adjusted logistic regression model.

**Table 3:** ORs and 95% CIs for the risk of incident HCC based on baseline serum AFP level and the level of AFP after genetic correction (per 10 ng/mL increase) in elderly Chinese people

Model	OR	95% CI	*P*
AFP level			
Model 1	4.70	2.48–8.90	<0.001
Model 2	4.55	2.34–8.85	<0.001
Model 3	5.04	2.46–10.30	<0.001
Genetic corrected AFP level			
Model 1	5.05	2.58–9.91	<0.001
Model 2	4.85	2.41–7.79	<0.001
Model 3	5.34	2.57–11.13	<0.001

Model 1, univariate model.

Model 2, adjusted for age, gender, smoking status, drinking status.

Model 3, adjusted for the variables in model 2 plus education, marriage, BMI, physical activity, family history of cancer.

AFP, alpha‐fetoprotein; BMI, body mass index; HCC, primary hepatocellular carcinoma; OR, odds ratio; 95% CI, 95% confidence interval.

Table 4 represented the associations between serum AFP levels corrected with genetic effects and risk of HCC by subgroups. As shown, the risk of HCC based on serum AFP levels after adjustment of genetic effects was higher in people with a history of smoking compared to those without a smoking history (OR = 2.15, 95% CI = 1.42–3.26; *P *<* *0.001 vs. OR = 1.19, 95% CI = 1.10–1.30; *P *<* *0.001). A same result appeared in the participants with or without a history of drinking, the OR for risk of HCC was higher in the subjects with a drinking history (OR = 2.03, 95% CI = 1.39–2.95; *P *<* *0.001 vs. OR = 1.20, 95% CI = 1.10–1.31; *P *<* *0.001). The similar situation was also seen in other subgroups of people with different gender, age, and BMI.

**Table 4:** ORs (95% CIs) for the risk of HCC based on baseline serum AFP after genetic correction, by subgroups

Subgroups	OR	95% CI	*P*
Gender^a^			
Male	2.57	1.42–4.65	0.002
Female	1.58	0.16–16.08	0.700
Age group^b^			
<62	1.76	1.03–3.00	0.039
≥62	5.05	2.18–11.67	<0.001
BMI group^b^			
<24	1.28	0.57–2.90	0.550
≥24	10.19	4.19–24.80	<0.001
Smoking status^b^			
Ever	2.15	1.42–3.26	<0.001
Never	1.19	1.10–1.30	<0.001
Drinking status^b^			
Ever	2.03	1.39–2.95	<0.001
Never	1.20	1.10–1.31	<0.001

AFP, alpha‐fetoprotein; BMI, body mass index; HCC, primary hepatocellular carcinoma; OR, odds ratio; 95% CI, 95% confidence interval.

a Adjusted for age, smoking status, drinking status, education, marriage, BMI, physical activity, family history of cancer.

b Adjusted for the other variables.

### Discriminatory ability of HCC for serum AFP level and genetic corrected AFP level

In order to calculate the discriminatory ability on the risk of HCC, the area under the ROC curve (AUC) was performed for the original serum AFP level and the AFP level with genetic correction. The discriminatory ability of HCC for serum AFP level after adjustment of genetic effects was higher compared with the original AFP level. With the effect of genetic correction, the AUC for the AFP level was 0.726 (95% CI: 0.652–0.799) with a sensitivity of 75.0% and a specificity of 66.3%. With compared to the genetic corrected AFP level, the original AFP level had an AUC of 0.611 (95% CI: 0.528–0.695) with a sensitivity of 61.5% and a specificity of 56.9%. The inclusion of sequence variants associated with AFP level increased the discriminatory ability by 18.8 percentage points (*P *=* *0.009).

## Discussion

This is the first study to assess the predictive value of genetic corrected AFP levels on HCC risk in a prospective cohort. Our finding shows that the effect of genetic correction could improve prediction efficiency of AFP levels for primary hepatocellular carcinoma risk in the elderly Chinese population. For the AUC to estimate the discriminatory ability on the risk of HCC, the genetic correction of AFP levels had an AUC value of 0.726 compared with the original AFP levels, an increase of 18.8%. The results of AUC analysis indicated that the prediction accuracy of genetic corrected AFP level for HCC was improved when genetic correction was taken application to the AFP level. This study suggests that the risk prediction performance of serum AFP level for HCC risk could be improved with the effect of genetic correction in the elderly Chinese population.

AFP is a kind of tumor antigen with single chain oncofetal glycoprotein approximately 70,000 Daltons in molecular weight and frequently unregulated in HCC 24. It has been demonstrated that physiological serum levels of AFP can exhibit a dose‐dependent growth‐regulatory activity toward developing cells or sensitive tumor 25, 26. On the other hand, high serum levels of AFP in HCC are associated with more aggressive tumor behavior and increased anaplasia, and are used as an indicator to monitor progression and metastasis of HCC 27, 28. The functional relationship between the *AFP* locus and AFP levels remains to be explored. *AFP* may protect hepatoma cells from immune surveillance by enhancing lymphocyte apoptosis and inhibiting hepatoma cell apoptosis 29. The regulation of *AFP* expression at the transcriptional level is complex. High AFP expression in human hepatoma cells is considered to be a key factor in promoting cancer cell survival in vivo 30, 31. It was found that AFP could affect the expression and proliferation of hepatoma cells expressing *AFP* receptors on the cell membrane 32, 33. The AFP receptor can also regulate tumor cell growth and induce activation of multiple signal transduction pathways 34. So AFP levels were taken as an important biomarker in the HCC diagnosis and treatment.

Although serum AFP level is widely used for HCC screening, its predictive value for HCC risk is currently limited. Several studies have explored the potential for AFP levels for HCC prediction and found the results were unsatisfactory 35, 36. The inter‐individual variation of serum AFP levels was found a long time ago 37. Some studies have also found that genetic variations can affect individual levels of serum AFP 38, 39. Studies have reported that a large proportion of the variability in AFP levels is due to heredity factors 40, 41. And our previous finding of a genome‐wide association study also demonstrated that the genetic variants have essential effect on the serum levels of AFP in Chinese. Although the underlying biological mechanism of these SNPs on serum AFP remains to be explored, the individual AFP levels could be estimated by these genetic variants. The SNP rs12506899 is located in the intron of *AFP* gene, however, it was found to be high linked with rs6834059, which is located at the transcription factor binding site of *AFP* gene and may affect the expression of *AFP*. On the other hand, there was no significant association between rs12506899 and the HCC risk in the present study. It indicated that the rs12506899 has impact on serum AFP level and further improves the prediction efficiency of AFP level on HCC risk. This situation was also seen in SNP rs2251844.

However, there are still certain limitations of this study that should be considered. First, only elderly people were enrolled in this study, and therefore it may not reflect the utility of AFP in the general population. All the subjects in this study came from Dongfeng automobile enterprise, including not only locals but also the people from every area in China. Therefore, such a conclusion could be drawn with a certain representative. Second, only the SNPs associated with AFP levels for genetic correction were taken into consideration. In effect, there may be other genetic variants associated with AFP levels besides SNPs. In consideration of other genetic variants more than SNPs in the future will help to learn the true role of AFP levels with genetic correction on the prediction efficiency for cancer risk. Although there are some shortcomings, our study still has important significance that cannot be ignored for further prediction of HCC, showing the prediction efficiency of genetic correction for serum AFP level on HCC risk. These limitations may be addressed when unidentified genetic variants which are associated with AFP are identified in the future. Further analysis with a longer follow‐up need to be implemented to confirm the role of serum biomarker as a predictive indicator for clinic application.

## Conclusions

In conclusion, we have identified that the significance in the prediction of primary hepatocellular carcinoma could be improved by adjusting the serum AFP levels with genetic effects. The genetic correction effect on serum AFP should be considered in the clinic application of such tumor biomarkers. Further studies of genetic effects on treatment and prognosis are warranted in order to further investigate the role of this tumor biomarker in the future.

## Ethical Approval

All the procedures performed in this study included human participants and experiments were according to the ethical standards of national research committee and in accordance with the 1964 Helsinki declaration. The human experimental protocols in this study were approved by the Medical Ethics Committee of the School of Public Health, Tongji Medical College, and Dongfeng General Hospital. Informed consent was obtained from all individual participants included in the study.

## Conflict of Interest

None declared.
